# Cloning, Bioinformatics Analysis, and Preparation of Polyclonal Antibody for Duck Cellular Inhibitor of Apoptosis Protein 1

**DOI:** 10.3390/vetsci13080830

**Published:** 2026-08-19

**Authors:** Sheng Yang, Yikun Zhu, Xin Wang, Yufei Huang

**Affiliations:** 1 College of Veterinary Medicine, Yangzhou University, Yangzhou 225009, China; 2 Jiangsu Co-Innovation Center for Prevention and Control of Important Animal Infectious Diseases and Zoonoses, Yangzhou 225009, China

**Keywords:** cellular inhibitor of apoptosis protein 1, molecular cloning, polyclonal antibody, duck

## Abstract

Cellular inhibitor of apoptosis protein 1 (cIAP1) plays a crucial role in the apoptotic process. This study involved gene cloning, protein expression, bioinformatics analysis, and the preparation of a specific polyclonal antibody. First, the coding sequence of the duck *cIAP1* was amplified by PCR and sequenced. Bioinformatics prediction indicated that this protein is a stable hydrophilic protein with no signal peptides and transmembrane domains. It contains characteristic BIR (bacterial IAP repeat) domains. The secondary structure consists of 43% random coils, 28% α-helix, and 8% β-strand, and its 3D structure is highly conserved compared with human and mouse cIAP1. Subsequently, the cIAP1 was cloned into the pET-28a vector. The recombinant His-tagged cIAP1 protein was successfully expressed in *Escherichia coli* BL21(DE3) upon induction and subsequently purified. Immunization of BALB/c mice with the purified protein emulsified in Freund’s adjuvant generated polyclonal antibodies. Indirect ELISA confirmed that the antibody titer exceeded 1:2,048,000. Additionally, Western blotting and immunofluorescence staining demonstrated that the antibody specifically recognized native duck cIAP1 protein without non-specific reactions with negative serum. This study successfully achieved prokaryotic expression of duck cIAP1 and produced a high-titer, highly specific mouse polyclonal antibody. This antibody is suitable for experiments such as Western blotting and immunofluorescence, providing important biological materials and a theoretical basis for further investigation of the anti-apoptotic mechanism of cIAP1 in major diseases of ducks.

## Simple Summary

Cellular inhibitor of apoptosis protein 1 is involved in apoptosis, intracellular signal transduction, and other biological processes, yet its function in ducks remains largely unexplored. In this study, the duck cellular inhibitor of apoptosis protein 1 was cloned and systematically analyzed by bioinformatics, and the soluble expression of the protein was produced in a prokaryotic expression system. Mice were immunized with the purified recombinant duck cellular inhibitor of apoptosis protein 1 as an immunogen, and the antibody with high specificity and sensitivity against duck cellular inhibitor of apoptosis protein 1 was obtained. This study offers a valuable tool for elucidating the roles and mechanisms of cIAP1 in duck development and disease.

## 1. Introduction

Apoptosis is a process of programmed cell death regulated by genes, which is one of the key mechanisms of maintaining homeostasis and has extensive and essential physiological and pathological significance. Apoptosis plays an important role in the response to viral infection. The cell death pathways induced by viral infections in poultry involve complex mechanisms, including apoptosis [1]. Viruses prevalent in ducks, such as duck viral enteritis virus (DVEV), duck hepatitis A virus (DHAV), and duck Tembusu virus (DTMUV), have also been reported to induce apoptosis in host cells [2,3,4]. Transcriptomic analysis of DTMUV-infected duck embryonic fibroblasts revealed dynamic changes in apoptosis-related genes (including anti-apoptotic and pro-apoptotic genes) [5]. However, in ducks, the molecular mechanisms underlying the interaction between the virus and host apoptosis regulators—particularly proteins of the inhabitor of apoptosis protein (IAP) family—remain poorly understood. Cellular inhibitor of apoptosis protein 1 (cIAP1) is a critical member of the IAP family, which can negatively regulate the process of apoptosis by directly inhibiting the activity of caspases; the precise regulation of this process is closely related to organismal development and disease [6,7]. Studies have shown that cIAP1 plays an important role in viral infection, cell death, innate immunity, and inflammatory response [8,9]. The function of cIAP1 in mammalian models such as humans and mice has been widely studied [10,11], but its role in poultry research is relatively limited. In particular, there is a lack of commercial antibodies that can specifically recognize duck cellular inhibitor of apoptosis protein 1 (DcIAP1). Therefore, the preparation of antibodies that specifically recognize DcIAP1 provides a key tool for studying its biological functions.

Here, the *DcIAP1* was cloned, and its molecular characteristics were revealed by bioinformatics analysis. The recombinant DcIAP1 protein was prepared by a prokaryotic expression system, and the antiserum was obtained by immunizing BALB/c mice. The titer, specificity, and sensitivity of the obtained polyclonal antibody were systematically identified by ELISA, Western blotting, and indirect immunofluorescence. These findings will help address the current paucity of research on duck cIAP1 antibodies and, in particular, provide key detection tools for further exploration of the biological functions of DcIAP1 in poultry.

## 2. Materials and Methods

### 2.1. Experimental Animals

Five adult female ducks and 20 SPF duck embryos were purchased from Nanjing Tegeili Planting Professional Cooperative (Nanjing, China); 4–6-week-old SPF BALB/c mice (female) were obtained from the Comparative Medicine Center of Yangzhou University (Yangzhou, China).

### 2.2. Cloning of CDS Region of the DcIAP1

Based on the predicted sequence of the *cIAP1* (XM_072032746.1) of duck (*Anas platyrhynchos*) in GenBank, a pair of specific amplification primers were designed by primer premier 5 software (F: 5′-ATGAACATAATGGAAAATAGCCCTTTC-3′, R: 5′- CCTTATGAAAGAAATGTACGAACTGTGCCC-3′) and synthesized by Beijing Tsingke Biotechnology Co., Ltd. (Beijing, China). Total RNA was extracted from duck brain tissue by a phenol chloroform extraction approach, and cDNA was obtained by reverse transcription (R433-01; Vazyme, Nanjing, China). The CDS sequence of the *DcIAP1* was amplified from the cDNA obtained by reverse transcription. The reaction procedure was: pre-denaturation at 95 °C for 5 min; denaturation at 95 °C for 30 s; annealing at 65 °C for 2 min; extension at 72 °C for 2 min, 40 cycles; extension at 72 °C for 5 min. PCR amplification products were subjected to 1% agarose gel electrophoresis, and the target band was cut at about 1800 bp. The recovered product, purified by the Gel Recovery Kit (GE706; Genesand, Beijing, China), is the complete CDS region of the *DcIAP1*. Furthermore, the CDS region was cloned into the pMD19-T cloning vector, and the resulting plasmid was extracted and sent to Nanjing Tsingke Biotech Co., Ltd. (Nanjing, China) for sequencing.

### 2.3. Bioinformatics Analysis of DcIAP1

The obtained CDS nucleotide sequences of the *DcIAP1* were translated into amino acid sequences, which were analyzed by the online software Smart (Simple Modular Architecture Research Tool, version 9.0, https://smart.embl.de/, accessed on 20 April 2026) with the default parameters (E-value threshold: 0.1) to identify its domains. The physicochemical properties, including molecular weight, theoretical isoelectric point, instability index, aliphatic index, and grand average of hydropathicity, were analyzed using Expasy ProtParam (https://web.expasy.org/protparam/, accessed on 20 April 2026). Signal peptide prediction was conducted using SignalP-6.0 (https://services.healthtech.dtu.dk/services/SignalP-6.0/, accessed on 20 April 2026) with the eukaryotic model and default cutoffs (probability threshold: 0.5). Hydrophilicity and hydrophobicity profiles were generated using the ProtScale tool (https://web.expasy.org/protscale/, accessed on 20 April 2026). Transmembrane helices were predicted using TMHMM-2.0 (https://services.healthtech.dtu.dk/services/TMHMM-2.0/, accessed on 20 April 2026) with the default hidden Markov model for eukaryotic proteins. Linear B-cell epitopes were predicted using the BepiPred-2.0 tool on the IEDB Analysis Resource website (http://tools.immuneepitope.org, accessed on 22 April 2026) with default parameters. The secondary structure was predicted using Phyre2 (Protein Homology/analog Y Recognition Engine V2.0, https://www.sbg.bio.ic.ac.uk/phyre2/, accessed on 23 April 2026) in the intensive mode, which uses multiple alignment and hidden Markov models to generate the most confident predictions. Homology modeling for the three-dimensional (3D) structure was performed using SWISS-MODEL (https://swissmodel.expasy.org/, accessed on 23 April 2026). The sequence identity threshold for template selection was set to ≥30%, and models with a Global Model Quality Estimation (GMQE) score > 0.7 and Qualitative Model Energy Analysis (QMEAN) score > 0.6 were selected for downstream analysis. Multiple sequence alignments of the full-length DcIAP1 with homologous sequences from other species, as well as the comparative alignment of the BIR domains, CARD, and RING domains among duck, mouse, and human, were performed using Clustal Omega (version 1.2.4, https://www.ebi.ac.uk/jdispatcher/msa/clustalo, accessed on 23 April 2026) with the default parameters. The alignments were visualized using MView (https://www.ebi.ac.uk/jdispatcher/msa/mview, accessed on 23 April 2026) and ESPript 3.0 (https://espript.ibcp.fr/, accessed on 23 April 2026), respectively. A phylogenetic tree was constructed using MEGA (Molecular Evolutionary Genetics Analysis, version 12.0) with the neighbor-joining (NJ) method, and the reliability of the tree topology was assessed by bootstrap analysis with 1000 replicates using the p-distance model. The protein–protein interaction network was constructed using the STRING database (version 12.0, https://string-db.org/, accessed on 23 April 2026) with the organism set to *Anas platyrhynchos*. The minimum required interaction score was set to 0.7 (medium confidence), and all other parameters were kept at the default values. The resulting network was exported for visualization and further analysis. The canonical IAP family domains (BIR (bacterial IAP repeat) domains, CARD (caspase activation and recruitment domain), and RING (really interesting new gene) domains) of cIAP1 from duck, mouse, and human species were compared through the online software Clustal Omega (https://www.ebi.ac.uk/jdispatcher/msa/clustalo, accessed on 25 April 2026), respectively, and visualized using ESPript 3 (https://espript.ibcp.fr/ESPript/ESPript/index.php, accessed on 25 April 2026). The 3D structure of the RING domain of the human cIAP1 protein was obtained from the PDB database (https://www.rcsb.org/, accessed on 25 April 2026) and served as a model to predict the RING domain of murine and duck cIAP1 proteins using SWISS-MODEL. The 3D structures were visualized using PyMOL software (version 4.6).

### 2.4. Construction of Prokaryotic Expression Vector pET28a-DcIAP1

To construct the recombinant plasmid pET28a-DcIAP1, the forward and reverse primers were designed to contain restriction sites at their 5′ ends (F: 5′-CGGGATCCATGAACATAATGGAAAATAGCCCTTTC-3′, R: 5′-CCCTCGAGTTATGAAAGAAATGTACGAACTGTGCCC-3′). The RNA extraction, reverse transcription, PCR product amplification, and gel recovery are the same as those in Section 2.1. The restriction endonucleases *BamH*I and *XHo*I (Thermo Fisher Scientific Inc., Waltham, MA, USA) were used to obtain the double-digested DcIAP1, which was then ligated with plasmid pET28a by T4 ligase (Takara Bio Inc., Kusatsu, Shiga, Japan) to construct the pET28a-DcIAP1 recombinant plasmid. The recombinant plasmid was transformed into *Escherichia coli* DH5α competent cells (CC96102; TOLOBIO, Shanghai, China), and a single uniform colony was selected and inoculated into LB liquid medium for culture. The positive clones were screened by PCR using the bacterial solution as a template. The PCR-positive monoclonal colonies were selected, and the recombinant plasmids were extracted using the Plasmid Extraction Kit (PE707; Genesand, Beijing, China) and sent to Nanjing Tsingke Biotech Co., Ltd. (Nanjing, China) for sequencing.

### 2.5. Optimization of Induction Conditions and Purification of DcIAP1 Recombinant Protein

The recombinant plasmid was transformed into *Escherichia coli* BL21 (DE3) (CC96107; TOLOBIO, Shanghai, China). To determine the optimal induction conditions, three parameters were independently optimized: induction temperature (16 °C, 25 °C, and 37 °C), isopropyl β-D-1-thiogalactopyranoside (IPTG, CI6621; Coolaber, Beijing, China) concentration (0.6, 0.8, and 1.0 mmol/L), and induction duration (12, 14, and 16 h). For each condition, bacteria were harvested and sonicated. An aliquot of the lysate was retained as the whole-cell lysate fraction, and the remaining lysate was centrifuged to obtain the supernatant and pellet fractions. All samples were analyzed by SDS-PAGE, followed by Coomassie Brilliant Blue staining. The optimal condition was defined as the combination that yielded the highest expression level of the target protein in the soluble fraction (supernatant) with minimal non-specific bands, as semi-quantitatively assessed by band intensity. The identified optimal conditions were then used for large-scale expression. The recombinant expression strain was cultured to a volume of 200 mL, disrupted using a homogenizer, and then centrifuged at 12,000 rpm at 4 °C for 20 min. The supernatant was collected and filtered through a 0.45 μm filter. Then, it was added to 2 mL of Ni-NTA beads and incubated in a shaking incubator in an ice bath for 2 h. The mixture was transferred to a 12 mL gravity chromatography column. The flow rate was adjusted using the roller clamp of a medical infusion set and maintained at 1 mL/min. The column passage was repeated twice to ensure thorough binding of the recombinant protein to the Ni column. A total of 10–15 times the column volume of elution buffer (50 mM NaH_2_PO_4_, 300 mM NaCl, 10 mM imidazole, PH 8.0) was added to remove impurity proteins, and the eluate was collected for SDS-PAGE electrophoresis. The target protein was then eluted stepwise with elution buffers containing increasing concentrations of imidazole: 50 mM, 100 mM, 200 mM, and 500 mM imidazole in 20 mM Tris-HCl and 500 mM NaCl; each elution fraction was collected and analyzed by SDS-PAGE. The fractions with the highest purity that contained the target protein were combined, dialyzed against phosphate-buffered saline (PBS, 137 mM NaCl, 2.7 mM KCl, 10 mM Na_2_HPO_4_, 1.8 mM KH_2_PO_4_, PH 7.4) at 4 °C, and then the solution was concentrated using urea. Subsequently, the concentration of protein was determined using the BCA method in preparation for subsequent immunological applications.

### 2.6. Preparation of Mouse Anti-Duck cIAP1 Protein Polyclonal Antibody

The purified DcIAP1 recombinant protein was diluted to the required concentration with PBS as the immunogen, fused with an equal amount of Freund’s complete adjuvant, emulsified, and subcutaneously immunized BALB/c mice. Freund’s complete adjuvant was used for the first immunization; Freund’s incomplete adjuvant was used for the second and third immunizations (P2036; P2031; Beyotime, Shanghai, China). Then, the purified protein was injected intraperitoneally. The protein dose for each mouse was 50 µg, and the interval between each immunization was 2 weeks. Blood samples were collected from the mice, and the serum was isolated to collect polyclonal antibodies.

### 2.7. Titer Detection of DcIAP1 Polyclonal Antibody by ELISA

Purified recombinant DcIAP1 protein was diluted to 2 µg/mL in coating buffer (0.05 M carbonate–bicarbonate buffer, pH 9.6) and coated onto 96-well ELISA plates (100 µL/well) overnight at 4 °C. The plates were washed three times with PBST (phosphate-buffered saline containing 0.05% Tween-20, pH 7.4) and blocked with 5% skim milk in PBST (200 µL/well) for 2 h at 37 °C. After three washes with PBST, serially diluted immune serum (starting from 1:1000 to 1:2,048,000 in PBST) was added (100 µL/well) and incubated for 1 h at 37 °C. Pre-immune serum from the same mice was used as a negative control, and PBST alone served as a blank control. Following three washes with PBST, HRP-labeled goat anti-mouse secondary antibody (RGAM001; Proteintech, Wuhan, China) diluted 1:5000 in PBST was added (100 µL/well) and incubated for 1 h at 37 °C. After five washes with PBST, 100 µL of TMB chromogenic solution (P0209; Beyotime, Shanghai, China) was added to each well and incubated in the dark for 15 min at room temperature. The reaction was terminated by adding 50 µL of 2 M H_2_SO_4_. The optical density at 450 nm (OD_450_) was measured using a microplate reader. The antibody titer was defined as the highest dilution at which the ratio of the OD_450_ of the immune serum to that of the pre-immune serum (P/N ratio) was ≥2.1. All assays were performed in triplicate.

### 2.8. The Application of DcIAP1 Polyclonal Antibody

To validate the specificity and sensitivity of the prepared polyclonal antibody, three complementary assays were performed: ELISA for titer determination, Western blotting for molecular weight verification and specificity testing in complex protein mixtures, and immunofluorescence for subcellular localization assessment. In all assays, pre-immune serum (collected from the same mice before immunization) was used as a negative control.

SPF duck embryo bodies were aseptically isolated on ice in PBS culture dishes containing 5% penicillin-streptomycin. After being chopped, they were digested with 0.25% trypsin for 15 min, passed successively through 100- and 200-mesh cell strainers, and then centrifuged at 1000 rpm for 10 min to collect the cells, resuspended in DMEM/F12, counted using a cell counter, and seeded at 5 × 10^5^ cells/mL into a 6-well cell culture plate. Duck embryo fibroblasts were seeded into 6-well plates and cultured until the cell density reached 70–80%. RIPA lysate (P0013B; Beyotime, Shanghai, China) and phenylmethylsulfonyl fluoride (PMSF) were added to lyse the cells and extract proteins. After denaturation, the cells were subjected to SDS-PAGE electrophoresis, membrane transfer, and blocking. The serum cIAP1 antibody was used as the primary antibody (1:5000) and incubated overnight at 4 °C. The next day, the membrane was rewarmed, washed, and incubated with HRP-labeled goat anti-mouse secondary antibody (1:5000) and then washed, colored, and exposed by a gel imaging system. The western blotting were employed to determine the specificity of the polyclonal antibodies and detect cIAP1 protein expressed in duck embryo fibroblasts.

Duck embryo fibroblasts were seeded onto cell-clinging pieces in 24-well plates and cultured until 70–80% confluence. The cells were fixed with 500 µL of 4% paraformaldehyde per well for 30 min at room temperature, followed by three washes with PBS (500 µL/well, 5 min each). Subsequently, the cells were permeabilized with 500 µL of 0.1% Triton X-100 in PBS for 10 min at room temperature and washed three times with PBS. Non-specific binding sites were blocked with 300 µL of 1% bovine serum albumin in PBS for 1 h at room temperature. After blocking, the cells were incubated overnight at 4 °C with 300 µL of anti-cIAP1 polyclonal antibody diluted 1:200 in PBS. Negative control wells were incubated with pre-immune mouse serum under the same conditions. After three washes with PBS (500 µL/well, 5 min each), the cells were incubated with 300 µL of FITC-conjugated goat anti-mouse secondary antibody (diluted 1:400 in PBS) for 1 h at 37 °C in the dark. Following three additional PBS washes, the nuclei were counterstained with 200 µL of DAPI (1 µg/mL) for 30 min at room temperature in the dark, and the cells were washed three times again with PBS. Finally, the coverslips were mounted with PBS and examined under an inverted fluorescence microscope, and images were captured.

## 3. Results

### 3.1. Cloning and Sequencing of CDS Region of the DcIAP1

**Figure 1:**
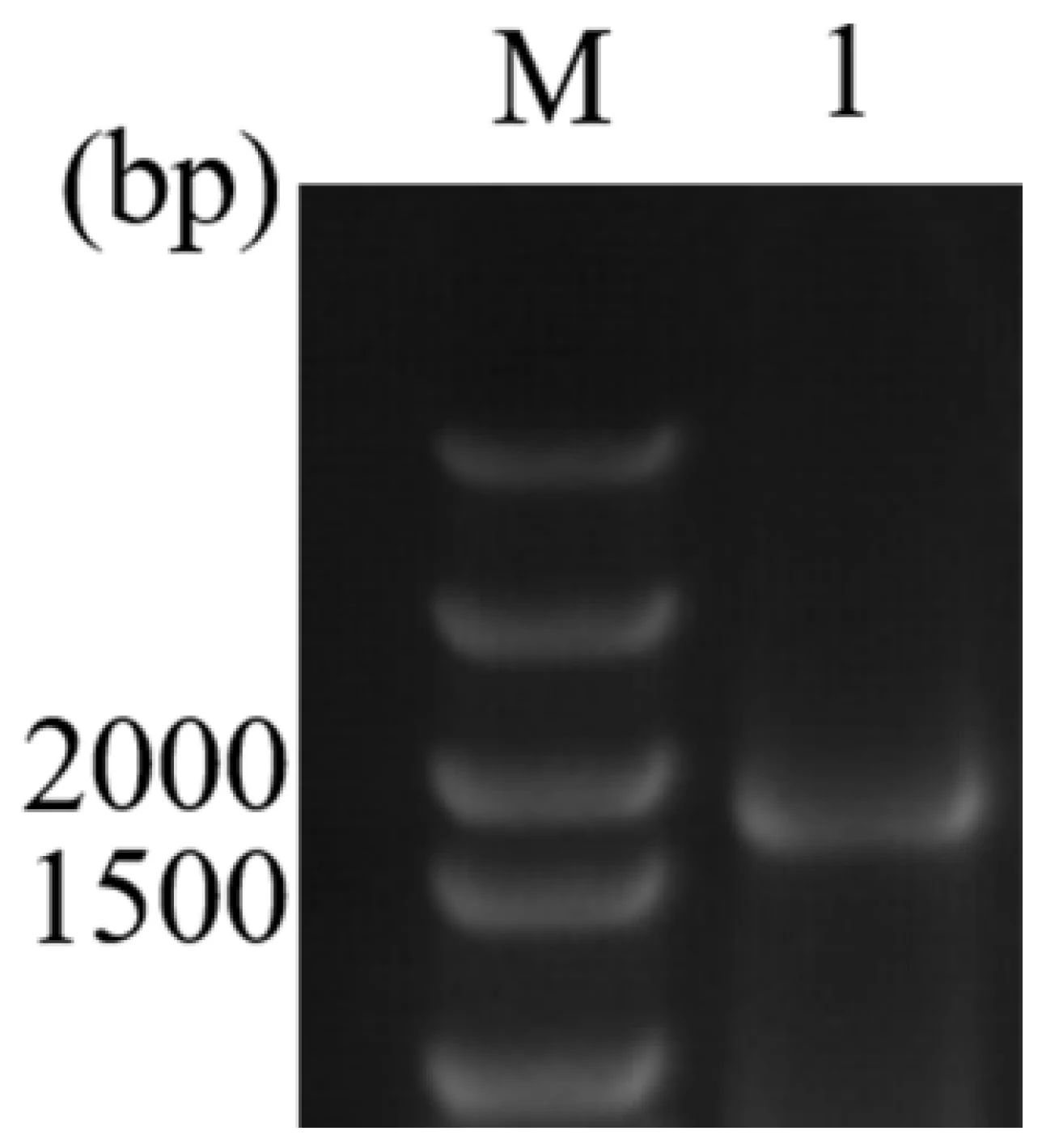
PCR amplification of the CDS region of *DcIAP1*.

**Figure 2:**
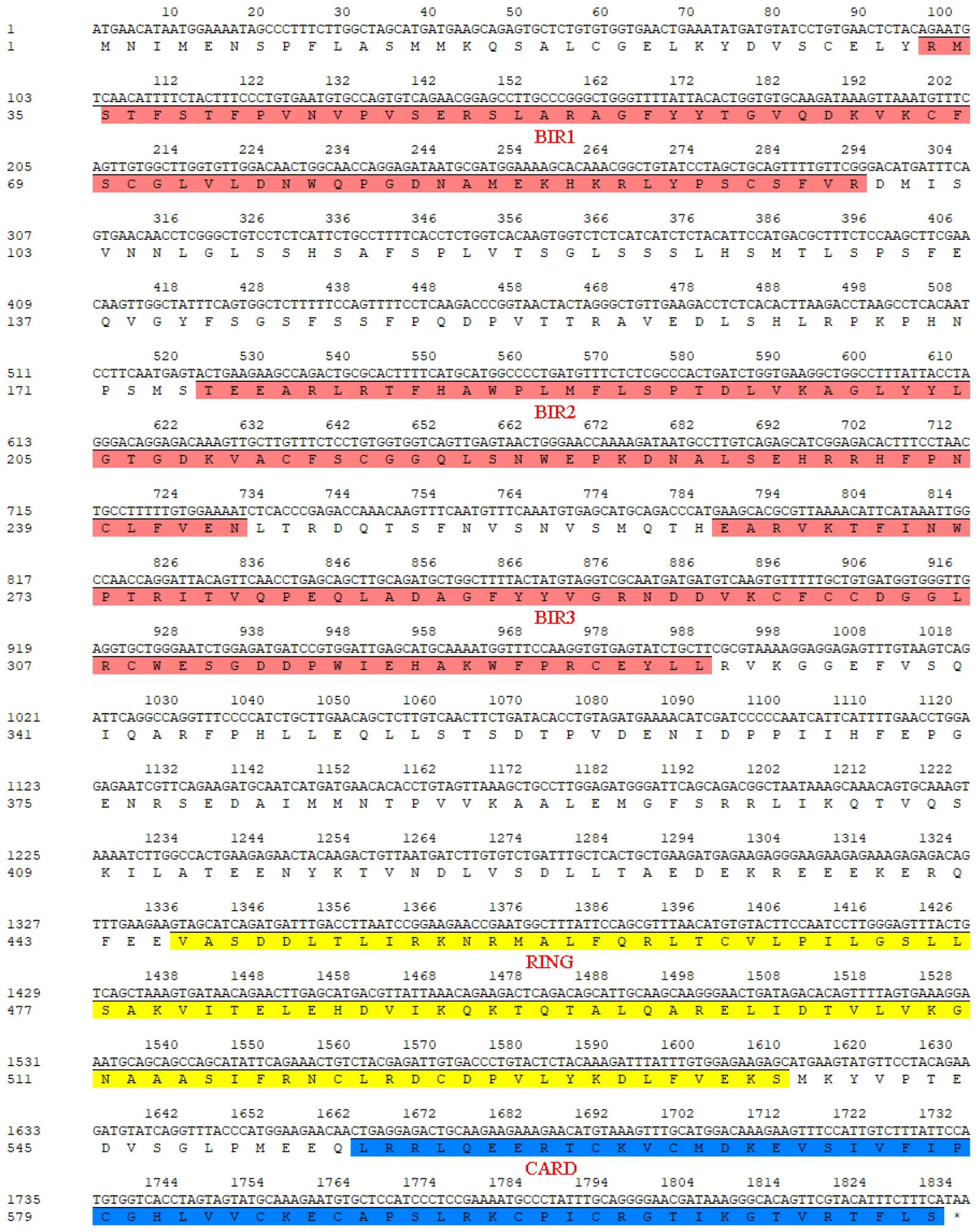
The cDNA and corresponding amino acid sequences of *DcIAP1*. The amino acid sequences marked in colors are domains; asterisks (*) indicate stop codons.

The results of PCR amplification of the CDS region of the *DcIAP1* (Figure 1) showed that the target protein band was consistent with the expected product size and was a single band. The CDS region of the *DcIAP1* is 1836 bp and encodes 611 amino acids. The translated protein sequences of the CDS region of the *DcIAP1* (Figure 2) are shown, and the classical protein domains were analyzed by SMART online software and are marked in colors, including BIR domains, RING domain, and CARD.

### 3.2. Bioinformatics Analysis of DcIAP1 Protein

**Figure 3:**
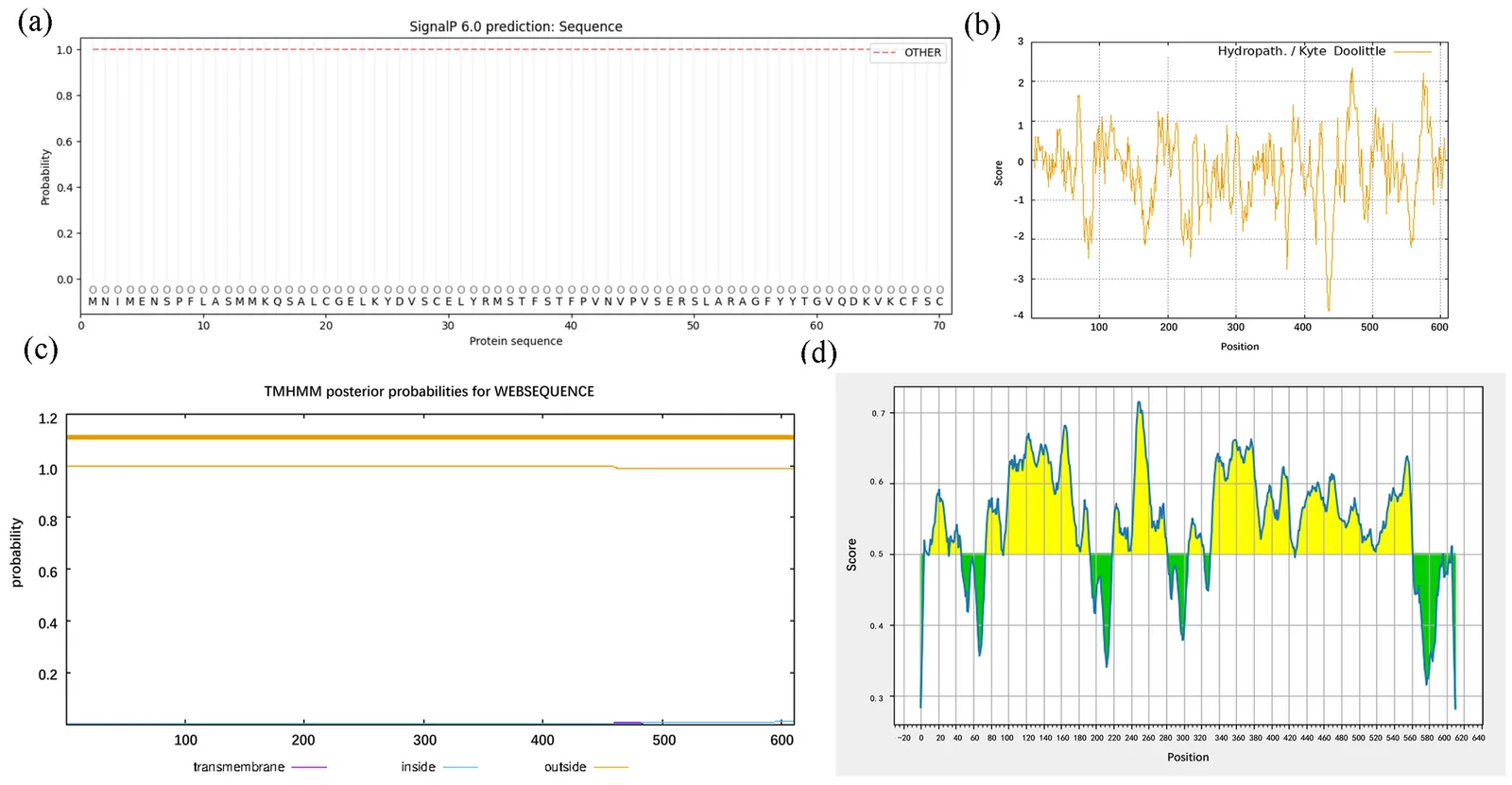
Bioinformatics analysis of the DcIAP1 protein. (**a**) Prediction of the signal peptide. (**b**) Prediction of hydrophilicity and hydrophobicity. (A high score indicates that the area is highly hydrophobic, while a low score indicates that it is highly hydrophilic). (**c**) The transmembrane region prediction. (The entire sequence is “outside,” indicating that there are no transmembrane regions). (**d**) Antigen epitope prediction. (The yellow sections represent predicted linear B-cell epitopes).

The results showed that the molecular weight of the DcIAP1 protein is 69.096 kDa, the theoretical isoelectric point is 5.94, and the molecular formula is C_3048_H_4795_N_833_O_917_S_41_ through ExPASY-Protparam online analysis software. The instability coefficient of the DcIAP1 protein is 52.44, the aliphatic index is 79.89, and the grand average of hydropathicity is −0.275. The signal peptide prediction results showed that the DcIAP1 protein has no signal peptide (Figure 3a); the hydrophilicity analysis showed that the hydrophilicity of the DcIAP1 protein near the C-terminus is high (Figure 3b); the DcIAP1 protein has no transmembrane region based on the analysis results of the online software TMHMM (Figure 3c). B cell epitope prediction revealed that amino acids 330–560 of the DcIAP1 protein are suitable for antigen sequence selection (Figure 3d).

**Figure 4:**
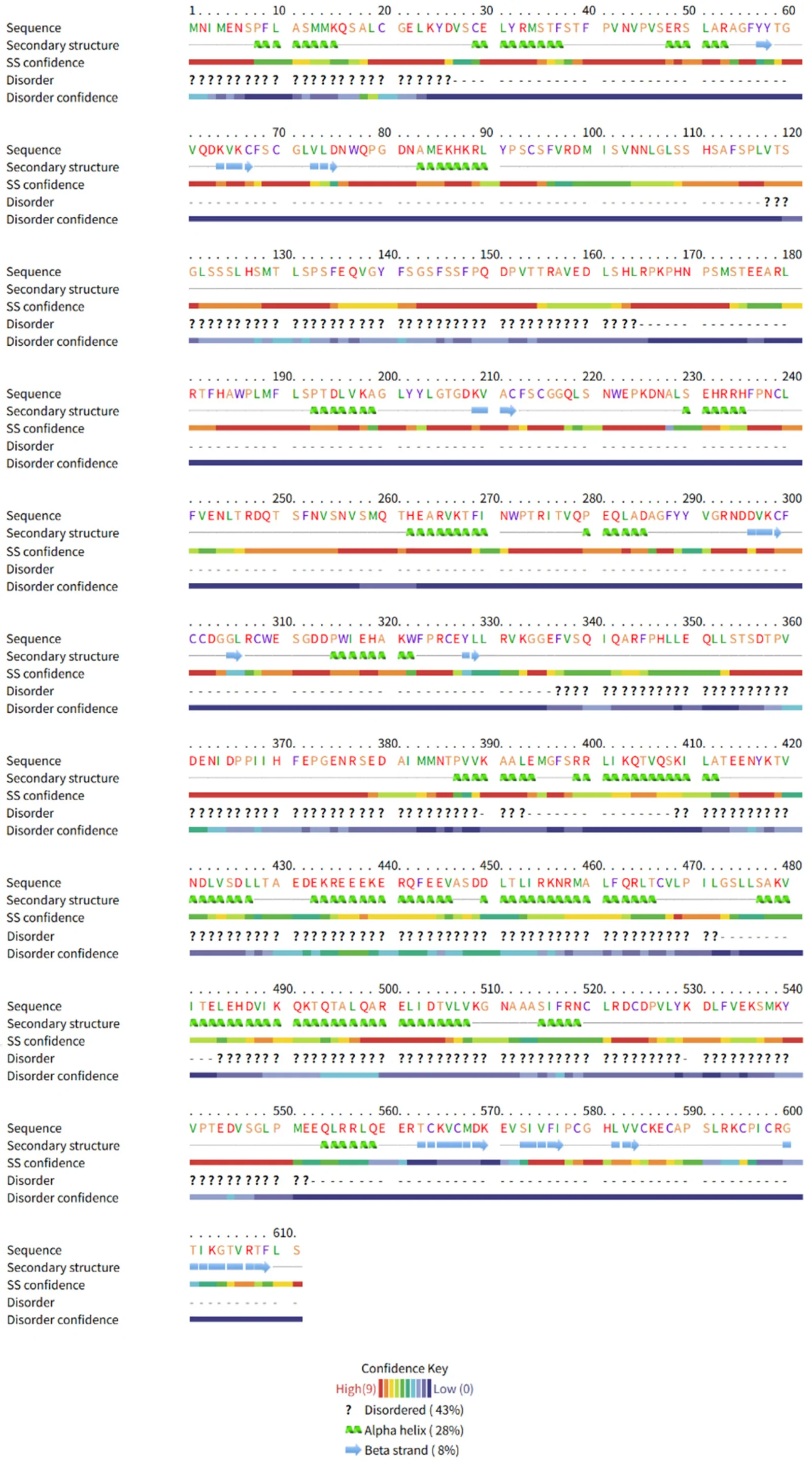
The secondary structure prediction of the DcIAP1 protein.

**Figure 5:**
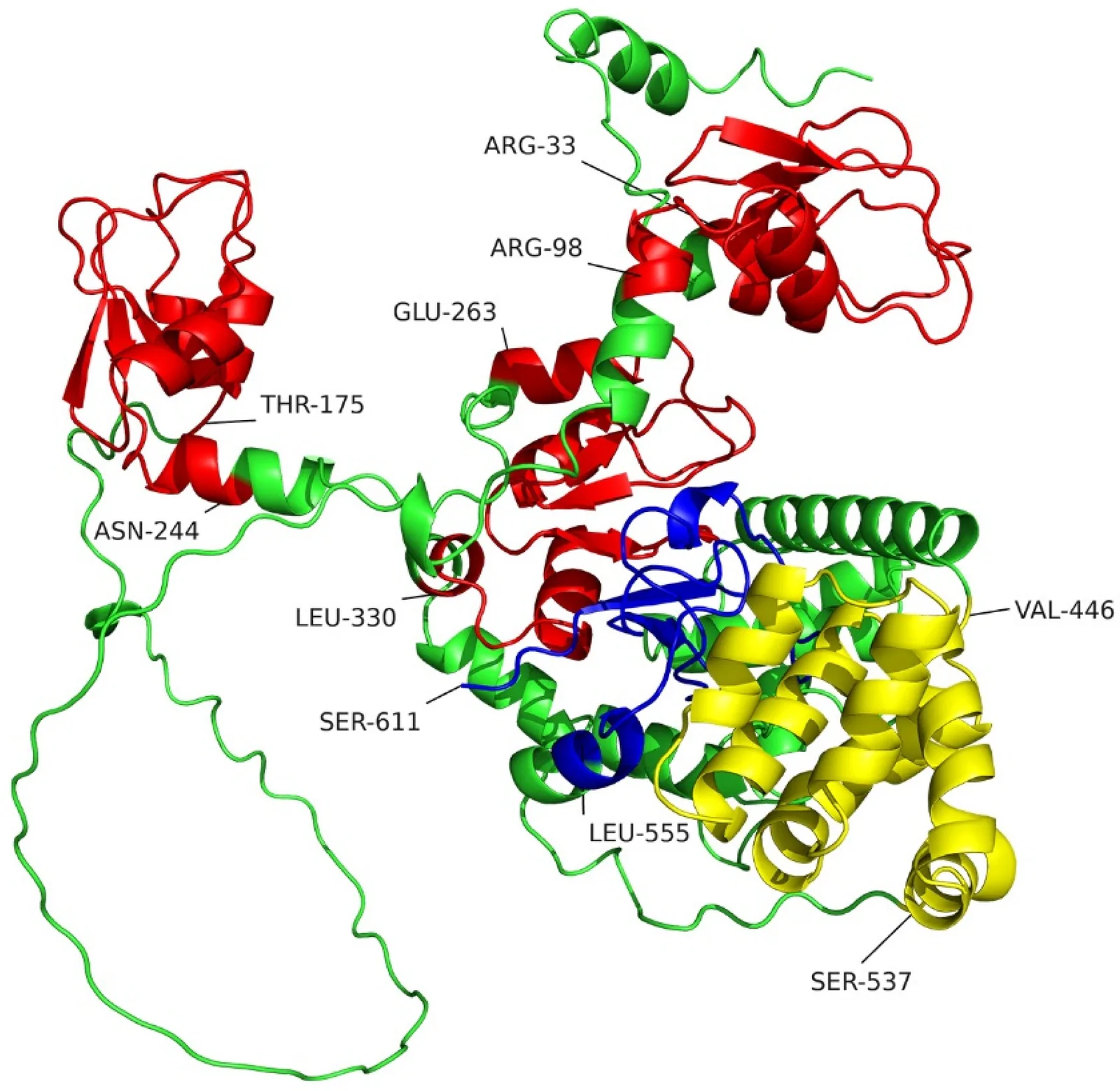
The 3D structure prediction. The red, yellow, and blue colors represent the typical motifs: BIR motifs (including BIR1 (ARG-33–ARG-98), BIR2 (THR-175–ASN-244), and BIR3 (GLU-263–LEU-330)), RING, and CARD motifs, respectively.

The secondary structure prediction of the DcIAP1 protein revealed that the green helices represent alpha helices (28%) and the blue arrows represent beta strands (8%) (Figure 4). The predicted 3D structure of the DcIAP1 protein was obtained via the online SWISS-MODEL software (Figure 5). The sequence consistency of the model reached 99% and GMQE > 0.7, indicating that the modeling quality is good and can be used for subsequent analysis. As presented in Figure 5, the red, yellow, and blue colors represent the classical motifs of the IAP protein family. Red is three typical BIR motif: BIR1 (ARG-33–ARG-98), BIR2 (THR-175–ASN-244), BIR3 (GLU-263–LEU-330). Yellow and blue represent the RING motif (VAL-446–SER-537) and CARD motif (ARG-556–LEU-610), respectively.

**Figure 6:**
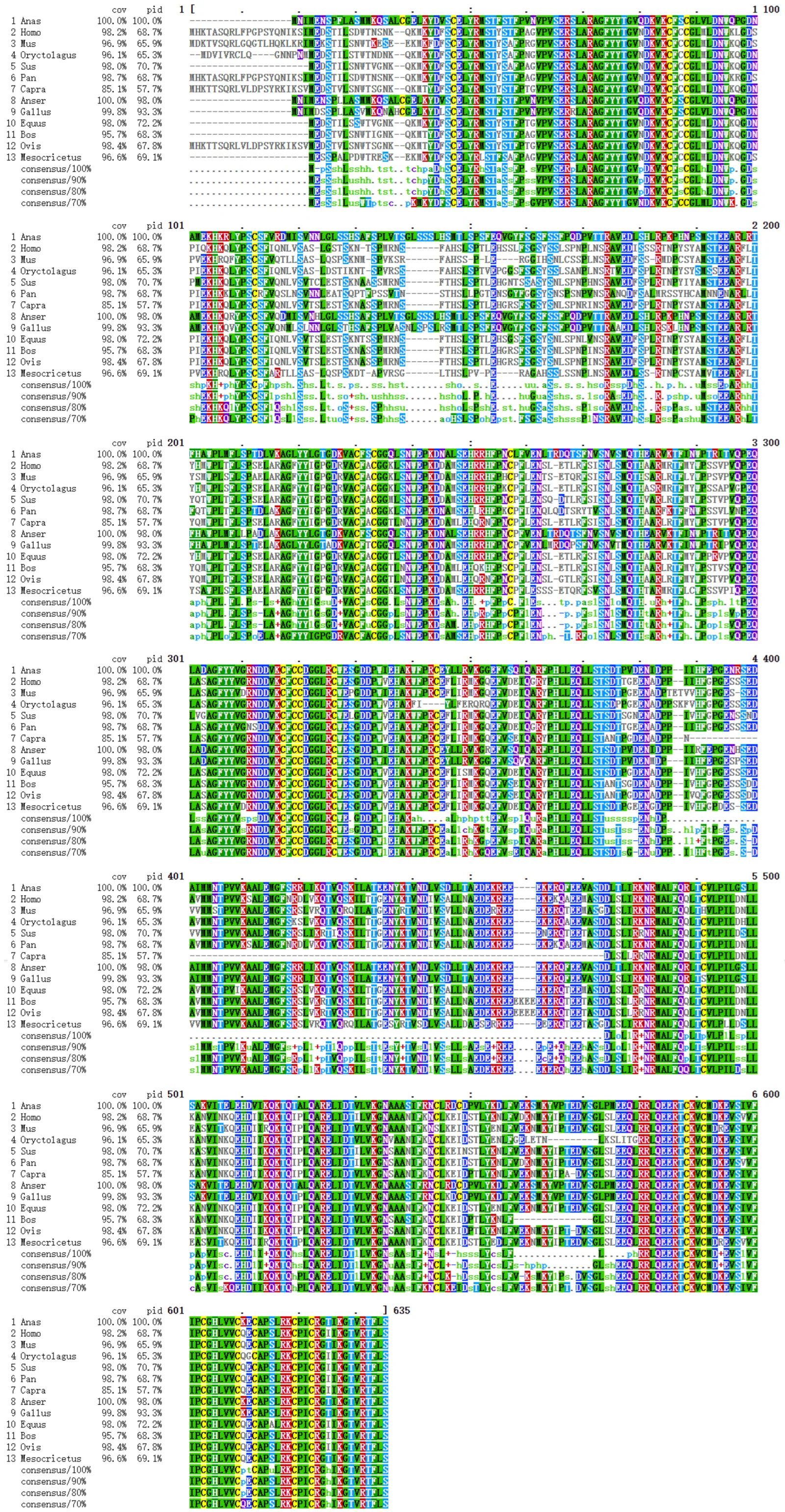
Multiple sequence alignments of the cIAP1 protein. Green represents hydrophobic residues (A, V, L, I, F, W, M, P, and G); purple represents amide-containing polar uncharged residues (Q, N); cyan represents hydroxyl-containing polar residues (S, T); blue represents negatively charged acidic residues (D, E); red represents positively charged basic residues (K, R); yellow represents cysteine (C); cov (coverage) refers to the percentage of the reference sequence covered by a given sequence in the alignment results; pid (percentage identity) refers to the percentage of identical amino acid residues between the sequence and the reference sequence within the aligned region; it is used to assess evolutionary relationships or functional conservation.

**Figure 7:**
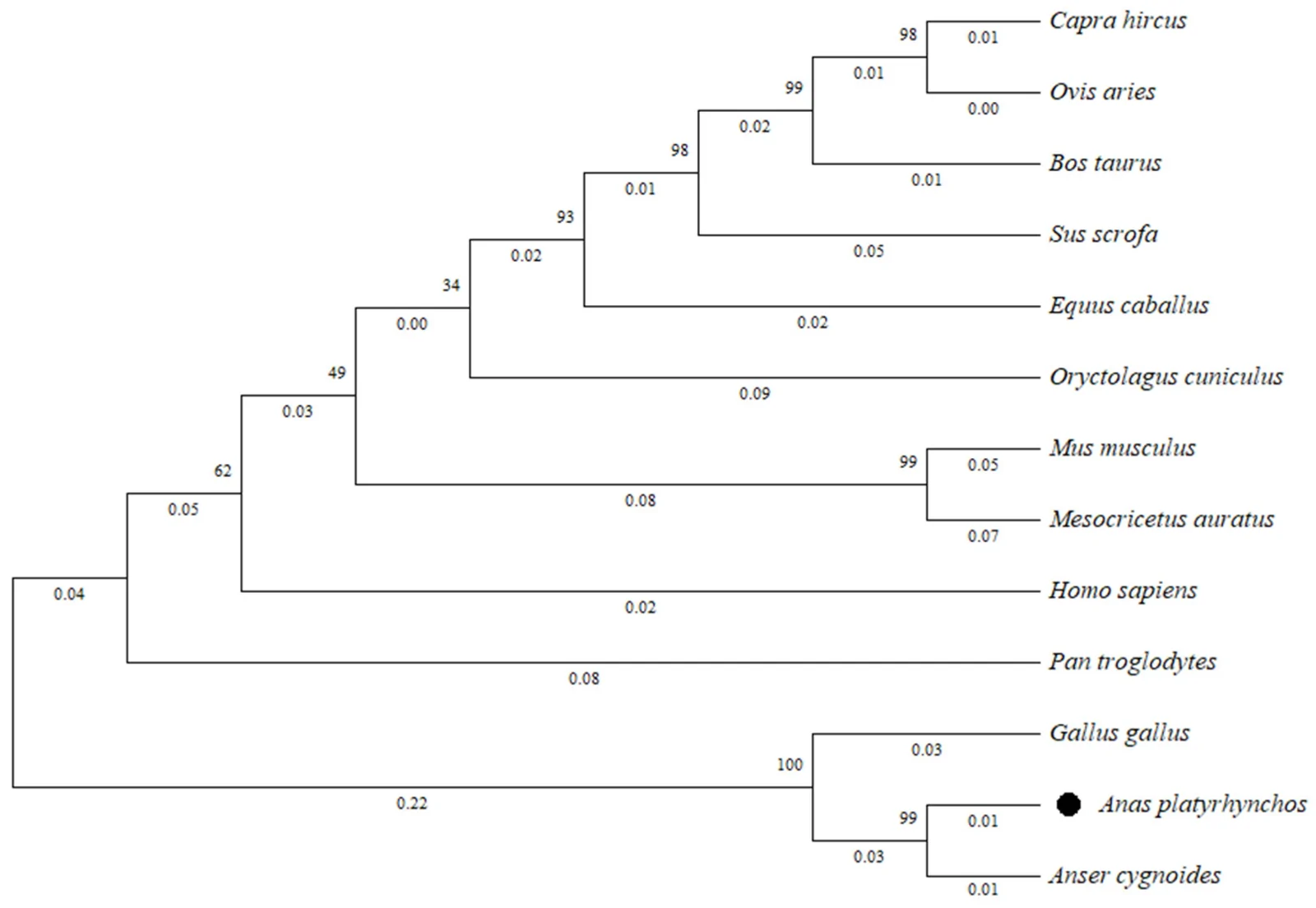
Phylogenetic tree of the cIAP1 protein.

The amino acid sequences of cIAP1 protein from 12 animals, including *Homo sapiens* (human), *Mus musculus* (mouse), *Oryctolagus cuniculus* (rabbit), *Sus scrofa* (pig), *Pan troglodytes* (chimpanzee), *Capra hircus* (goat), *Anser cygnoides* (goose), *Gallus gallus* (chicken), *Equus caballus* (horse), *Bos taurus* (cattle), *Ovis aries* (sheep), and *Mesocricetus auratus* (golden hamster) were obtained through the UniProt website, which were compared with those of DcIAP1. The results of multiple sequence alignment showed that DcIAP1 has high identity with the cIAP1 protein of other species, and the highest identity with the *Anser cygnoide* cIAP1 protein was 98.02%, followed by *Gallus gallus* with 93.22% (Table 1). The N-terminus of the DcIAP1 protein is quite different from that of other species, and the middle region and C-terminus are highly conserved (Figure 6). Phylogenetic analysis reveals that DcIAP1 has a shorter evolutionary distance and a closer phylogenetic affinity with *Anser cygnoides* and *Gallus gallus* (Figure 7).

**Figure 8:**
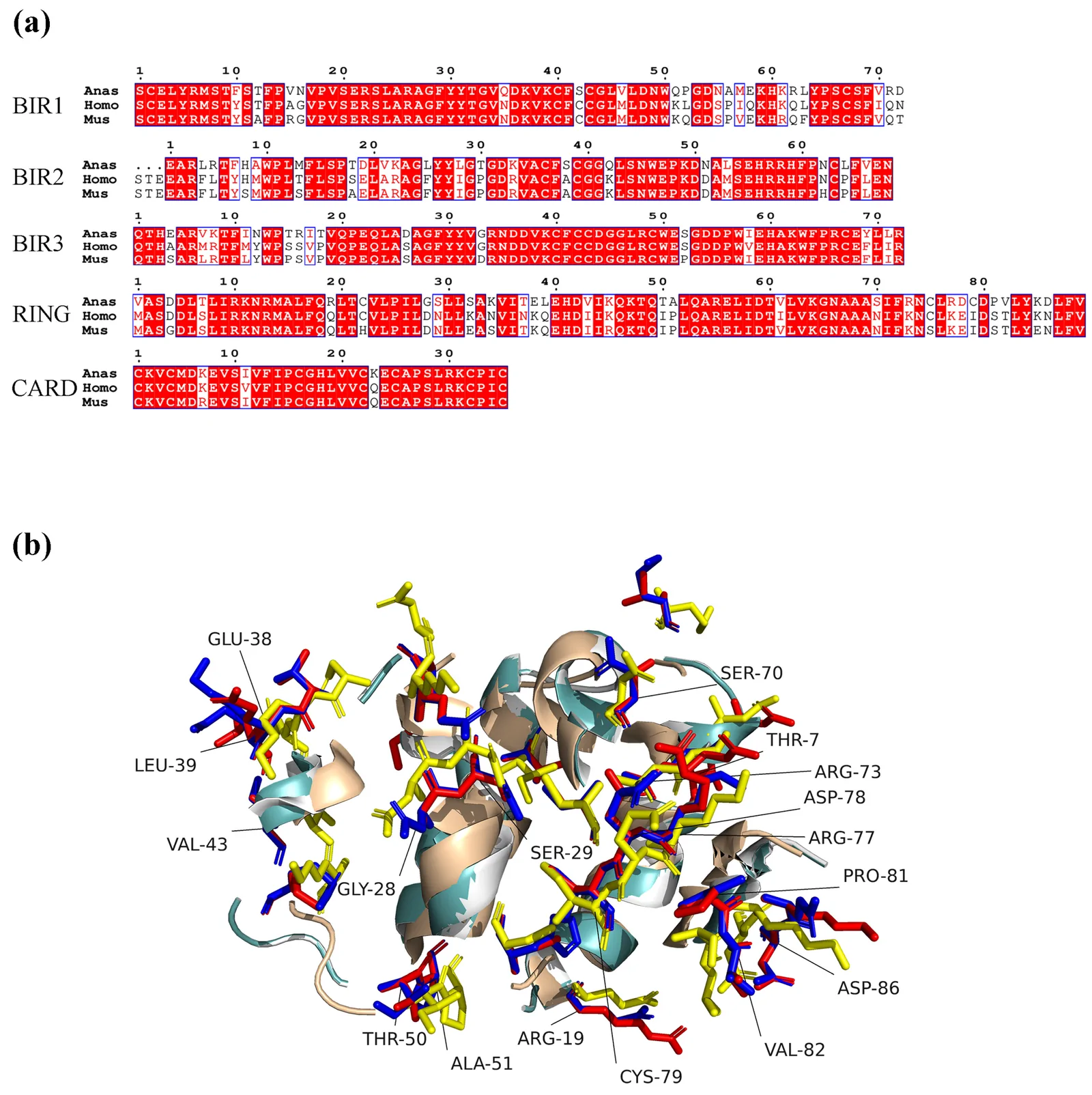
Comparison of classical domains of IAP proteins. (**a**) BIR domains and CARD of IAP proteins. (**b**) 3D structure of the RING domain. Red indicates amino acid residues from duck cIAP1, yellow indicates amino acid residues from human cIAP1, and blue indicates amino acid residues from mouse cIAP1.

The sequences of *Anas platyrhynchos* (duck), *Homo sapiens* (human), and *Mus musculus* (mouse) cIAP1 proteins were downloaded from GenBank. Their BIR domains, CARD, and RING domains were aligned by Clustal Omega online software and visualized by ESPript 3. The results (Figure 8a) showed that the BIR domains and CARD of DcIAP1 were conserved, especially at the core sites related to protein interaction. The alignment results of the 3D structure of the RING domain (Figure 8b) showed that the sequence differences are mainly located in the surface loop region.

**Figure 9:**
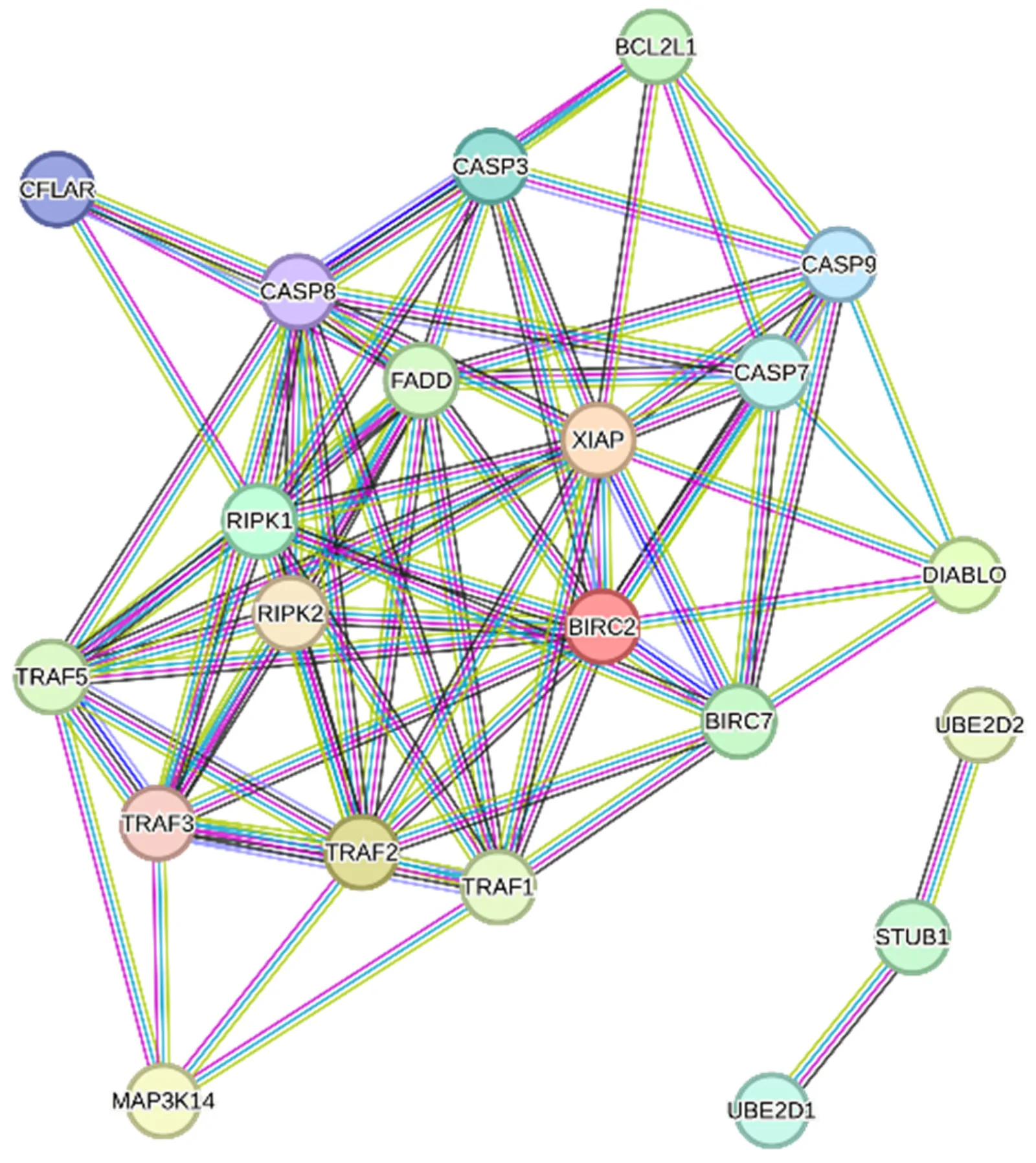
Protein–protein interaction network associated with DcIAP1. BIRC2 is an alternative name for cIAP1.

The cIAP1-related target proteins (relevance score > 2) were searched from Genecards, and the duck species was selected from the STRING database to construct a protein interaction network (high confidence = 0.700). The findings (Figure 9) showed that a total of 20 high-confidence interacting proteins were obtained. The network is highly connected, and the core nodes include cIAP1 (BIRC2) itself, TNF receptor-associated factor 2 (TRAF2), receptor-interacting serine/threonine kinase 1 (RIPK1), caspase 3 (CASP3), etc.

### 3.3. Construction, Expression, and Purification of Recombinant DcIAP1 Protein

**Figure 10:**
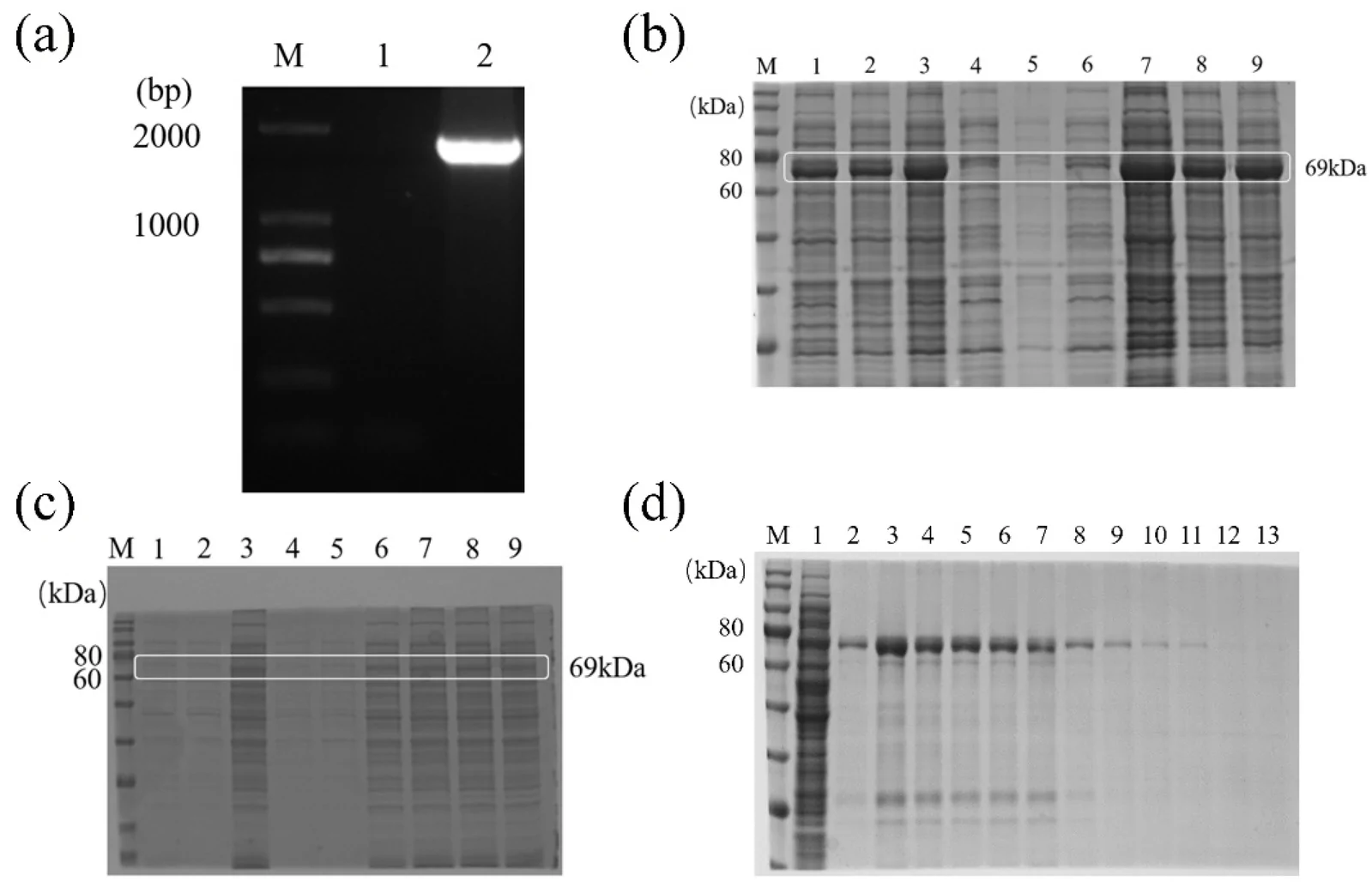
Construction, expression, and purification of recombinant DcIAP1 protein. (**a**) PCR results for the recombinant plasmid pET28a-DcIAP1. M, 2000 DNA marker; 1, negative control; 2, bacterial colony. (**b**) Optimal temperature of induction conditions for DcIAP1. M, 180 kDa protein marker; 1–3 represent the bands of DcIAP1 in the whole bacterial liquid at 37, 25, and 16 °C, respectively; 4–6 represent the bands of DcIAP1 in the bacterial supernatant at 37, 25, and 16 °C, respectively; 7–9 represent the bands of DcIAP1 in the bacterial precipitation at 37, 25, and 16 °C, respectively. (**c**) Optimal concentration of IPTG for induction conditions for DcIAP1 in the bacterial supernatant. M, 180 kDa protein marker; 1–3 represent the bands of DcIAP1 using 0.6 mmol/L IPTG at 12 h, 14 h, and 16 h, respectively; 4–6 represent the bands of DcIAP1 using 0.8 mmol/L IPTG at 12 h, 14 h, and 16 h, respectively; 7–9 represent the bands of DcIAP1 using 1 mmol/L IPTG at 12 h, 14 h, and 16 h, respectively. (**d**) Purification of DcIAP1 recombinant protein. M, 180 kDa protein marker; 1, flow-through solution; 2–13, imidazole eluent.

The ligation products (pET28a-DcIAP1 recombinant plasmid) obtained in Section 2.4 were transformed into *Escherichia coli* DH5α competent cells, and the PCR results of bacterial solution were obtained after cloning (Figure 10a). To find the optimal conditions of protein yield, SDS-PAGE was performed, and the results (Figure 10b,c) indicated that the expression of supernatant was the highest when induced by 1 mmol/L IPTG at 16 °C. Therefore, the optimal induction conditions for the recombinant *Escherichia coli* BL21(DE3) harboring pET28a-cIAP1 were 16 °C and 1 mmol/L IPTG for 16 h.

The recombinant protein was purified using Ni-NTA beads 6FF, and the flow-through solution and eluate were collected for SDS-PAGE analysis. The results showed that there was a single protein band at 69 kDa (Figure 10d), indicating that the recombinant protein with high purity was obtained by purification.

### 3.4. Determination of Serum Titer and Application of DcIAP1 Polyclonal Antibody

**Figure 11:**
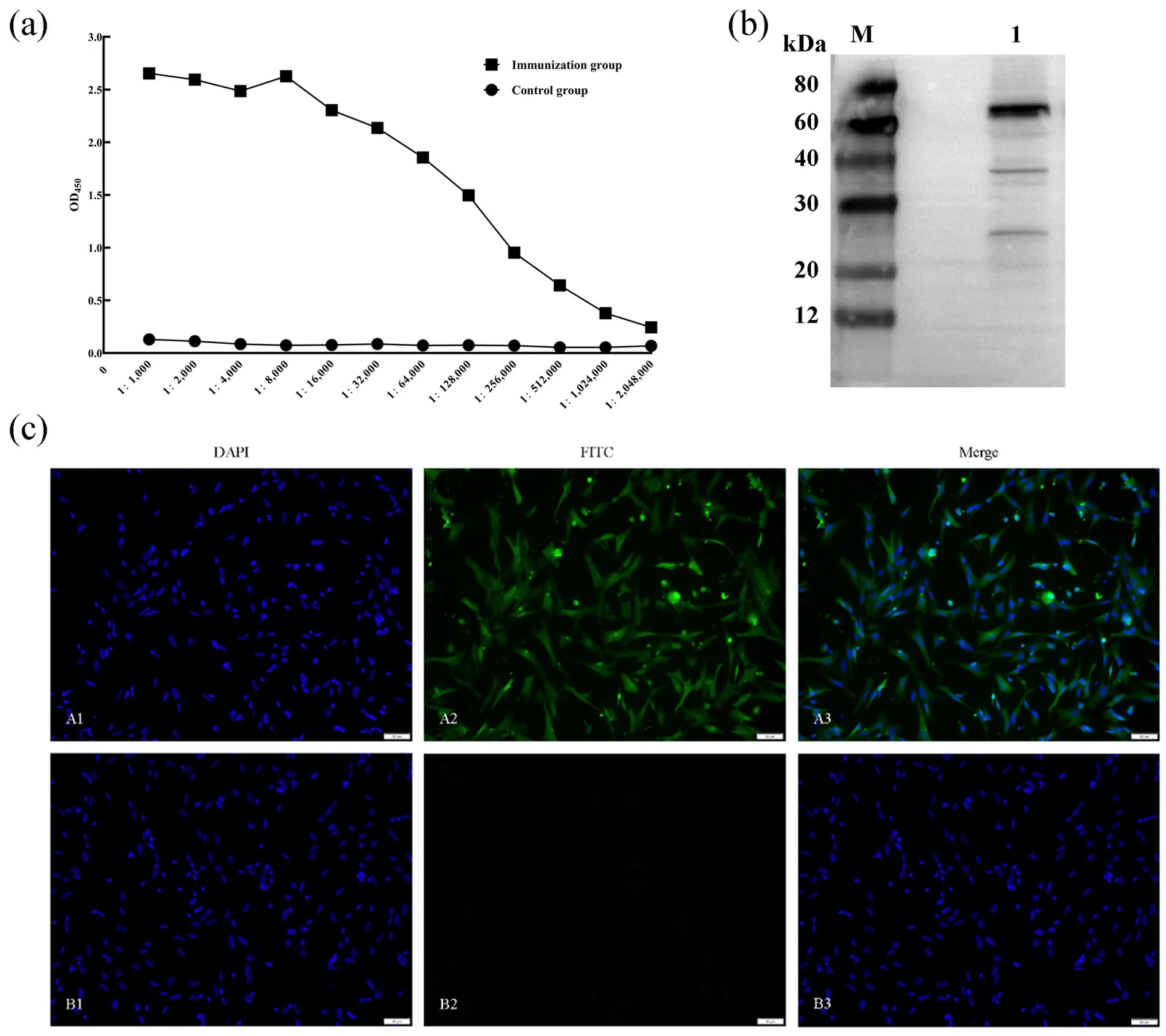
Determination of serum titer and application of DcIAP1 polyclonal antibody. (**a**) Assay of cIAP1 polyclonal antibody titers (the original OD values and P/N ratios can be found in Table S1). (**b**) Western blotting. M, 80 kDa protein marker; 1, cIAP1 in the duck fibroblast protein sample (the original Western blotting pictures can be found in Figure S1). (**c**) Identification of DcIAP1 polyclonal antibody by indirect immunofluorescence. (**A1**–**A3**), polyclonal antibody; (**B1**–**B3**), negative serum; (**A1**,**B1**), nuclei stained with DAPI; (**A2**,**B2**), cytoplasm stained with FITC; (**A3**) Merge of DAPI (blue) and FITC (green) showing DcIAP1 localization in the cytoplasm. (**B3**) Merge of DAPI (blue) and FITC (green) for negative serum control, showing no specific staining.

The DcIAP1 polyclonal antibody was diluted to 1:2,048,000 and coated on an ELISA plate, and the antibody titer was determined by a microplate reader. The negative control was the serum of non-immunized mice. It was found that the titer of DcIAP1 polyclonal antibody was greater than 1:2,048,000 (Figure 11a).

Duck fibroblast protein samples were identified with a 1:5000 dilution of DcIAP1 polyclonal antibody. It was found that the polyclonal antibody could recognize endogenous DcIAP1, and the protein band size was about 69 kDa, consistent with the expected DcIAP1 size (Figure 11b).

The duck embryo fibroblasts were inoculated onto the coverslips; the serum DcIAP1 antibody was used as the primary antibody (1:200), and the FITC-labeled sheep anti-mouse fluorescent secondary antibody (1:400) was used for indirect immunofluorescence. The findings (Figure 11c) showed that duck embryo fibroblasts incubated with DcIAP1 polyclonal antibody exhibited bright and extensive green fluorescence in the cytoplasm, with over 80% of cells being positive and fluorescence intensity markedly above background. In contrast, fibroblasts incubated with negative serum displayed no detectable green fluorescence signal. These findings suggest that the DcIAP1 polyclonal antibody specifically recognizes native cIAP1 expressed in duck embryo fibroblasts with high selectivity.

## 4. Discussion

As an important member of the IAP family, cIAP1 is a key regulator of apoptosis, inflammatory responses, and innate immunity [10]. Its antagonist has shown good application value in the treatment of inflammation and cancer prevention [12,13,14]. Recent studies have elucidated that cIAP1 regulates cell death and tissue inflammation by modulating the receptor-interacting protein kinase 1 (RIPK1) [9]. In addition to the downstream effects, the stability and activity of cIAP1 are also subject to tight regulation at the post-translational level. For example, deubiquitination by USP36 has been shown to stabilize cIAP1 and thereby inhibit apoptosis [15]. Therefore, cIAP1 plays a crucial role in both apoptosis and inflammation and represents a potential drug target.

Relatively few studies have been conducted on the biological functions of DcIAP1 in poultry. Predicting and analyzing the structure of DcIAP1 through methods such as gene cloning and bioinformatics analysis will facilitate subsequent functional studies of DcIAP1. In this study, bioinformatics analysis revealed that DcIAP1 contains typical BIR domains, CARD, and RING domains. The BIR domains are known to mediate protein–protein interactions, including binding to TRAF2 for the regulation of the NF-κB signaling pathway [16,17]. Additionally, the BIR domains can also bind to the second mitochondria-derived activator of caspase to relieve caspase inhibition [18,19]. The CARD exerts inhibitory functions by limiting RING dimerization and E2 binding [20]. The RING domain possesses E3 ubiquitin ligase activity targeting itself and substrates such as TRAF2 and XIAP [21]. The BIR domains and CARD of DcIAP1 are evolutionarily conserved, suggesting that the basic functions of these domains—including caspase inhibition, TRAF binding, and ubiquitination—are similar in birds and mammals [22,23]. Multiple sequence alignment further showed that DcIAP1 shares more than 93% identity with poultry species (geese and chickens) and approximately 64–68% identity with mammals, implying possible avian-specific functional regulation mechanisms during evolution. Protein–protein interaction network predictions suggested that DcIAP1 may participate in apoptosis and inflammatory signaling through interactions with TRAF2, RIPK1, CASP3, and other IAP family members, but these bioinformatics-based predictions require direct experimental verification.

Polyclonal antibodies against avian proteins can be effectively produced through prokaryotic expression and an appropriate immunization protocol. In the present study, a switch from the pGEX-6P-1 vector to another expression vector, and a change of the host strain to Rosetta (DE3), were prompted by the low purification yield during expression optimization. However, this replacement did not produce the expected effect on protein yield. This failure may be attributed to the intrinsic properties of the target protein, codon usage bias, or incompatibility between the expression vector and host strain. Indirect ELISA showed that the polyclonal antibody titer reached 1:2,048,000, indicating its suitability for highly diluted detection applications. Western blotting analysis confirmed that the antibody specifically recognized a protein band of approximately 69 kDa in duck embryo fibroblasts, consistent with the theoretical molecular weight of DcIAP1. The immunofluorescence results further demonstrated that the polyclonal antibody could specifically recognize endogenous DcIAP1 localized in the cytoplasm, consistent with the functional localization of cIAP1 as a cytoplasmic protein. Beyond basic detection, this antibody holds potential for studying DcIAP1 in duck viral infections. Several duck pathogens, such as DVEV, DHAV, and DTMUV, have been reported to regulate apoptotic pathways [2,3,4]. Given that DcIAP1 is an anti-apoptotic protein and its role during viral infection remains to be elucidated, this antibody provides a valuable tool for in-depth studies of the interactions between DcIAP1 and viral proteins.

Specific antibodies targeting duck cIAP1 have been rarely reported. The polyclonal antibody prepared in this study has been validated in duck samples by ELISA, Western blotting, and immunofluorescence. However, the polyclonal antibody may exhibit batch-to-batch variability. Importantly, its applicability in immunoprecipitation, immunohistochemistry, or neutralization assays has not been tested and requires future validation. Thus, this antibody offers a useful tool for basic detection applications, but its broader utility remains to be established.

## 5. Conclusions

In summary, this study cloned the duck cIAP1 and characterized its protein structure, revealing conserved domains and species-specific sequence features that warrant further functional exploration. A mouse polyclonal antibody against DcIAP1 was successfully generated and validated for specific detection of endogenous DcIAP1 in duck cells, providing a practical tool for basic expression studies in poultry. Future work should assess its utility in additional applications (e.g., immunoprecipitation or immunohistochemistry) and investigate the functional roles of DcIAP1 in duck disease models.

## Figures and Tables

**Table 1:** Comparison of the cIAP1 protein identity between duck and other species.

Species Name	Identity/%	Coverage/%
*Anser cygnoides*	98.02	100.0
*Gallus gallus*	93.22	99.8
*Equus caballus*	68.86	98.0
*Sus scrofa*	66.79	98.0
*Pan troglodytes*	66.59	98.7
*Homo sapiens*	66.39	98.2
*Bos taurus*	66.32	95.7
*Ovis aries*	65.99	98.4
*Mesocricetus auratus*	65.71	96.6
*Mus musculus*	64.48	96.9
*Capra hircus*	64.06	85.1
*Oryctolagus cuniculus*	63.13	96.1

## Data Availability

The data presented in this study are available on request from the corresponding author due to privacy.

## Supplementary Materials

The following supporting information can be downloaded at: https://www.mdpi.com/article/10.3390/vetsci13080830/s1, Figure S1: DcIAP1 polyclonal antibody; Table S1: OD values and P/N ratios measured by indirect ELISA.

## References

[1] Sun Y., Liu K. (2024). Mechanistic insights into Influenza A virus-induced cell death and emerging treatment strategies. Vet. Sci..

[2] Zhang Y., Wang X., Hu A., Wu Y., Zhang P., Yang X., Wen Z., Wen M. (2021). Duck enteritis virus infection suppresses viability and induces apoptosis and endoplasmic reticulum stress in duck embryo fibroblast cells via the regulation of Ca(2). J. Vet. Med. Sci..

[3] Sheng X.D., Zhang W.P., Zhang Q.R., Gu C.Q., Hu X.Y., Cheng G.F. (2014). Apoptosis induction in duck tissues during duck hepatitis A virus type 1 infection. Poult. Sci..

[4] Huang Y., Zhang Y., Yang S., Shi Y., Chu X., Ahmed N., Wu J., Chen Q. (2024). Tembusu virus induced apoptosis in vacuolate spermatogenic cells is mediated by Cytc-mediated mitochondrial apoptotic signaling pathway. Theriogenology.

[5] Pan Y., Cheng A., Zhang X., Wang M., Chen S., Zhu D., Liu M., Zhao X., Yang Q., Wu Y. (2020). Transcriptome analysis of duck embryo fibroblasts for the dynamic response to duck tembusu virus infection and dual regulation of apoptosis genes. Aging.

[6] Elsässer A., Suzuki K., Lorenz-Meyer S., Bode C., Schaper J. (2001). The role of apoptosis in myocardial ischemia: A critical appraisal. Basic Res. Cardiol..

[7] Miller S.A., Briglin A. (1996). Apoptosis removes chick embryo tail gut and remnant of the primitive streak. Dev. Dyn..

[8] Liu X., Chen J., Li Z., Gao N., Zhang G. (2024). CIAP1/2 can regulate the inflammatory response and lung injury induced by apoptosis in septic rats. J. Investig. Med..

[9] Schorn F., Werthenbach J.P., Hoffmann M., Daoud M., Stachelscheid J., Schiffmann L.M., Hildebrandt X., Lyu S.I., Peltzer N., Quaas A. (2023). cIAPs control RIPK1 kinase activity-dependent and -independent cell death and tissue inflammation. EMBO J..

[10] Zadoroznyj A., Dubrez L. (2022). Cytoplasmic and nuclear functions of cIAP1. Biomolecules.

[11] Akizuki Y., Morita M., Mori Y., Kaiho-Soma A., Dixit S., Endo A., Shimogawa M., Hayashi G., Naito M., Okamoto A. (2023). cIAP1-based degraders induce degradation via branched ubiquitin architectures. Nat. Chem. Biol..

[12] Bai L., Smith D.C., Wang S. (2014). Small-molecule SMAC mimetics as new cancer therapeutics. Pharmacol. Ther..

[13] Zhang J., Webster J.D., Dugger D.L., Goncharov T., Roose-Girma M., Hung J., Kwon Y.C., Vucic D., Newton K., Dixit V.M. (2019). Ubiquitin ligases cIAP1 and cIAP2 limit cell death to prevent inflammation. Cell Rep..

[14] Smolewski P., Robak T. (2011). Inhibitors of apoptosis proteins (IAPs) as potential molecular targets for therapy of hematological malignancies. Curr. Mol. Med..

[15] Gao B., Qiao Y., Zhu S., Yang N., Zou S.-S., Liu Y.-J., Chen J. (2024). USP36 inhibits apoptosis by deubiquitinating cIAP1 and survivin in colorectal cancer cells. J. Biol. Chem..

[16] Lin S.-C., Huang Y., Lo Y.-C., Lu M., Wu H. (2007). Crystal structure of the BIR1 domain of XIAP in two crystal forms. J. Mol. Biol..

[17] Mahoney D.J., Cheung H.H., Mrad R.L., Plenchette S., Simard C., Enwere E., Arora V., Mak T.W., Lacasse E.C., Waring J. (2008). Both cIAP1 and cIAP2 regulate TNFalpha-mediated NF-kappaB activation. Proc. Natl. Acad. Sci. USA.

[18] Dueber E.C., Schoeffler A.J., Lingel A., Elliott J.M., Fedorova A.V., Giannetti A.M., Zobel K., Maurer B., Varfolomeev E., Wu P. (2011). Antagonists induce a conformational change in cIAP1 that promotes autoubiquitination. Science.

[19] Hennessy E.J., Adam A., Aquila B.M., Castriotta L.M., Cook D., Hattersley M., Hird A.W., Huntington C., Kamhi V.M., Laing N.M. (2013). Discovery of a novel class of dimeric Smac mimetics as potent IAP antagonists resulting in a clinical candidate for the treatment of cancer (AZD5582). J. Med. Chem..

[20] Oetjen K.A., Duckett C.S. (2011). Identifying the trigger of c-IAPs: Structural and functional characterization of CARD-mediated modulation of ubiquitin ligase activity. Mol. Cell.

[21] Cheung H.H., Plenchette S., Kern C.J., Mahoney D.J., Korneluk R.G. (2008). The RING domain of cIAP1 mediates the degradation of RING-bearing inhibitor of apoptosis proteins by distinct pathways. Mol. Biol. Cell.

[22] Verhagen A.M., Coulson E.J., Vaux D.L. (2001). Inhibitor of apoptosis proteins and their relatives: IAPs and other BIRPs. Genome Biol..

[23] Lopez J., John S.W., Tenev T., Rautureau G.J., Hinds M.G., Francalanci F., Wilson R., Broemer M., Santoro M.M., Day C.L. (2011). CARD-mediated autoinhibition of cIAP1’s E3 ligase activity suppresses cell proliferation and migration. Mol. Cell.

