# Supplementary material for: Cloning, Bioinformatics Analysis, and Preparation of Polyclonal Antibody for Duck Cellular Inhibitor of Apoptosis Protein 1
Source: Vet Sci. 2026 Aug 19;13(8):830. doi: 10.3390/vetsci13080830 (PMC13517590; doi:10.3390/vetsci13080830)

Figure S1. DcIAP1 polyclonal antibody.

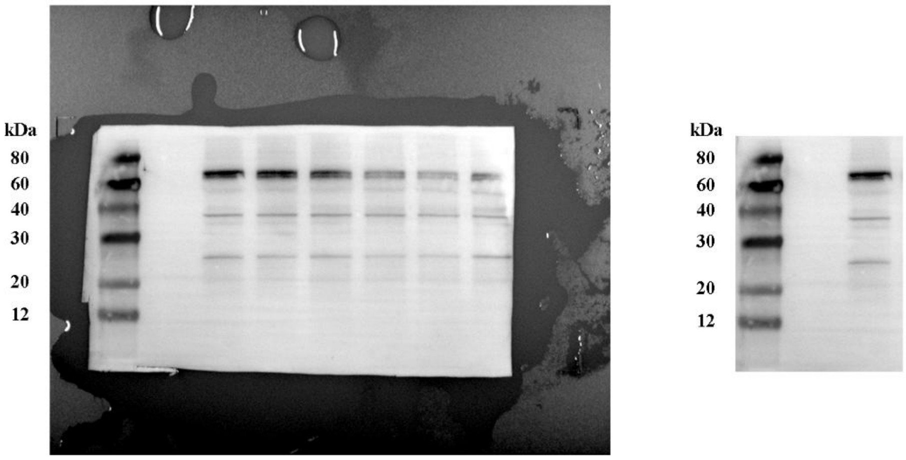

Supplement: Supplementary file 1 [file vetsci-13-00830-s001.zip › Figure S1.pdf]
