# Supplementary material for: Cloning, Bioinformatics Analysis, and Preparation of Polyclonal Antibody for Duck Cellular Inhibitor of Apoptosis Protein 1
Source: Vet Sci. 2026 Aug 19;13(8):830. doi: 10.3390/vetsci13080830 (PMC13517590; doi:10.3390/vetsci13080830)
Supplement: Supplementary file 1 [file vetsci-13-00830-s001.zip › Table S1.pdf]

Table S1. OD values and P/N ratios measured by indirect ELISA.

Dilution Ratio	OD_{450} (control group)	OD_{450} (immunization group)	P/N
1:1000	0.130	2.654	20.415
1:2000	0.113	2.594	22.956
1:4000	0.085	2.485	29.235
1:8000	0.073	2.626	35.973
1:16000	0.077	2.304	29.922
1:32000	0.086	2.136	24.837
1:64000	0.072	1.856	25.778
1:128000	0.075	1.497	19.960
1:256000	0.071	0.952	13.408
1:512000	0.054	0.642	11.889
1:1024000	0.054	0.379	7.019
1:2048000	0.068	0.244	3.588
